# Feasibility and effectiveness of a telephone-based social support intervention for informal caregivers of people with dementia: Study protocol of the TALKING TIME project

**DOI:** 10.1186/s12913-017-2231-2

**Published:** 2017-04-17

**Authors:** Martin Berwig, Martin Nikolaus Dichter, Bernd Albers, Katharina Wermke, Diana Trutschel, Swantje Seismann-Petersen, Margareta Halek

**Affiliations:** 1 Department of Psychiatry and Psychotherapy, Medical Faculty, Leipzig University, Semmelweisstraße 10, 04103 Leipzig, Germany; 2 German Center for Neurodegenerative Diseases (DZNE), Stockumer Straße 12, 58453 Witten, Germany; 3 School of Nursing Science, Faculty of Health, Witten/Herdecke University, Stockumer Straße 12, 58453 Witten, Germany

**Keywords:** Dementia, Informal caregivers, Telephone-based intervention, Psycho-social intervention, Social support, Health-related quality of life

## Abstract

**Background:**

Caring for people with dementia at home requires a significant amount of time, organization, and commitment. Therefore, informal caregivers, mainly relatives, of people with dementia often feel a high burden. Although on-site support groups are known to have positive effects on the subjective well-being (SWB) and perceived social support of informal caregivers, there are cases in which relatives have either no time or no opportunity to leave the person alone or in which there are no support groups nearby. The TALKING TIME project aims to close this supply gap by providing structured telephone-based support groups in Germany for the first time. International studies have shown benefits for informal caregivers.

**Methods:**

The TALKING TIME study is a randomized controlled trial. The effects of the 3-month TALKING TIME intervention will be compared with those of a control group without intervention at two time points (baseline = T_0_, after 3 months = T_1_). The control group will receive the TALKING TIME intervention after T_1_. With a planned sample size of 88 participants, the study is powered to detect an estimated effect size of 0.70 for psychological quality of life, considering an α of 0.05 (two-sided), a power of 80%. Caregivers are informal caregivers who are eligible if they are 18 years of age or older and have cared for a person with diagnosed dementia for at least four hours, four days per week, in the past six months. The exclusion criteria are psychiatric disorders of the informal caregiver. The primary outcome is the mental component summary of the SF-12 rated by informal caregivers. The secondary outcomes for informal caregivers are the physical component summary of the SF-12, the Perceived Social Support Caregiver Scale (SSCS) score, and the Caregiver Reaction Scale (CRS) score. The secondary outcome for care recipients is the Neuropsychiatric Inventory (NPI-Q). For the process evaluation, different quantitative and qualitative data sources will be collected to address reach, fidelity, dosage and context.

**Discussion:**

The results will provide further information on the effectiveness and optimization of telephone-based support groups for informal caregivers of people with dementia, which can help guide the further development of effective telephone-based social support group interventions.

**Trial registration:**

Clinical Trials: NCT02806583, June 9, 2016

## Background

More than one million people with dementia live in Germany, and it is expected that this number will increase from 1 to 1.5 million currently to 3.0 million people by 2050 [1]. Globally, and particularly in Germany, the family remains the cornerstone of care for older people [2], and therefore, informal (family) caregivers take over much of the responsibility of caring for and supporting people with dementia. More than two-thirds (70%) of care recipients who live at home are cared for predominantly by partners and children [3]. Almost half of these care recipients are also people with dementia [4]. Supporting and caring for people with dementia are time-consuming and are associated with significant personal engagement and day-to-day management. Therefore, informal caregivers of people with dementia often show a higher stress level than that of caregivers of physically frail elderly people [5, 6] and have an increased risk of becoming physically and mentally ill [6]. These negative consequences can be prevented by interventions that improve the individual coping abilities and perceived social support of informal caregivers. The individual and social resources of the informal caregiver are crucial factors that have a moderating influence on the care process and are important components in explaining the association between primary stressors (e.g., support with (instrumental) activities of daily living, emotional support, practical caregiving tasks, challenging behaviors) and secondary stressors (e.g., conflicts between personal needs and care requirements, role conflicts) and the long-term consequences for the health and subjective well-being (SWB) of the informal caregivers [7].

The benefits of psycho-educative interventions, cognitive-behavioral therapy, counseling/case management, general support (e.g., support groups), and respite interventions for informal caregivers of people with dementia with respect to burden and depressive symptoms, ability/knowledge, and SWB have been demonstrated in several studies [6, 8].

In this context, social support interventions are of particular interest because due to the growing cost pressure in the healthcare system, informal caregivers are increasingly asked to rely on their own networks for assistance and support [2]. Several different interventions have been developed to increase caregiver support [9]. One of the major services is caregiver support group interventions that target the exchange of experiences among informal caregivers. Informal caregivers are experts in caring for their relatives with dementia, and therefore, they can support each other and give valuable advice. Group participants experience the opportunity for personal exchange with other people who are in a comparably stressful situation as being emotionally relieving and supportive [10, 11]. There is evidence that psycho-educative groups have a positive influence on psychological well-being and the experience of burden, particularly if an active exchange of experience among participants occurs [6, 8, 10].

Despite these positive empirical findings, the utilization of support groups in general is particularly low [12–14]. For informal caregivers, the management of care for people with dementia and the poor accessibility of support groups, particularly in rural areas, is an obstacle to attending a group activity outside the home [15–17]. For these reasons, in recent years, so-called remote interventions for informal caregivers of people with dementia have been developed, providing social support by online networks, chat forums, videophone, or telephone and thus overcoming the dependency on location [9]. Several studies concerning the effectiveness of these remote social support interventions are available. However, the results regarding social support as an outcome are heterogeneous. For example, videoconferencing interventions show some indications of effects favoring the intervention, whereas for telephone support interventions, no effects have been found [9].

This lack of effectiveness may be explained by the application of very different instruments that capture the diverse domains of the social support concept [9].

In relation to other outcomes, social support interventions have yielded promising results. For example, videoconferencing interventions demonstrate improved self-efficacy and reduced feelings of burden, distress, and depression [9, 18–20]. Telephone-based group interventions show effects on quality of life, feelings of burden, caregiver symptomatology, and the depressivity of informal caregivers [9, 11, 21–24].

To date, however, remote social support interventions, which offer the possibility of exchange among several participants, are very rare in German-speaking areas. One German pilot study that evaluated an internet-based training program for informal caregivers of people with dementia revealed a wide range of barriers to the use of internet technology by informal caregivers, e.g., a weak broadband network in rural areas, worries of the informal caregivers regarding computer technology and worries related to the intervention procedures in general [25].

**Fig. 1:**
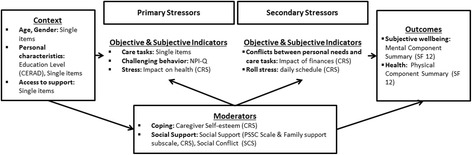
Outcome model of the TALKING TIME study Legend. Outcome model of the Talking Time study based on the models of informal caregiver’s stress process by Peralin et al. 1990 and Gutzman et al. 2005. CERAD = Consortium to Establish a Registry for Alzheimer’s Disease; NPI-Q = Neuropsychiatric Inventory – Q; CRS = Caregiver Reaction Scale; PSSC = Perceived Social Support Caregiving Scale; SCS = Perceived Social Conflict Scale; HRQoL = Health related Quality of Life; SF-12 = General Health Survey Questionnaire Short Form 12

In this respect, telephone-based offers have great advantages: most people are used to telephones, which are available in most households in Germany; consequently, technological barriers are unlikely, even among the elderly. Surprisingly, to date, telephone-based support groups for informal caregivers of people with dementia are not available in Germany, and trials to investigate the effectiveness of such support groups are lacking. Therefore, the TALKING TIME project is the first investigation of the feasibility, effectiveness, and possible types of harm of telephone-based support groups. The study includes an evaluation of the intervention processes and intervention effectiveness. The outcome model is based on the models of the informal caregivers stress process [7, 26]. The model is summarized in simplified form in Fig. 1.

### Study aim and research question

The aims of this Medical Research Council (MRC) framework phase II trial [27] were translated into the following research question:

- 1.Does the TALKING TIME intervention positively affect the health-related quality of life (HRQoL) of informal caregivers of people with dementia, their perceived social support, and the reaction of the informal caregivers?

To detect possible types of intervention harm for care recipients, a second research question asks the following:

- 2.Does the TALKING TIME intervention affect the challenging behavior of people with dementia who are cared for by an informal caregiver?

Two research questions target the investigation of the study and intervention processes (process evaluation):

- 3.To what extent can the intervention be delivered as planned (degree of implementation)?
- 4.What are the facilitators of and barriers to the TALKING TIME intervention?

## Methods/Design

### Design

**Fig. 2:**
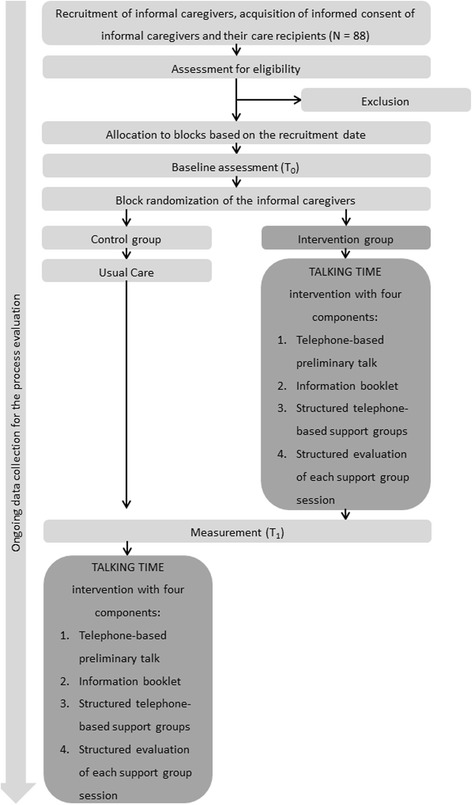
Study design

The evaluation of the TALKING TIME intervention is based on data of a two level design, where informal caregivers are nested within groups of eight persons. Informal caregivers will be randomized within a group into support and control group, four informal caregivers each (Fig. 2). Measurements are assessed at two measurement points resulting in repeated measurements of informal caregivers (Fig. 1). Computer-generated randomization lists will be used. The randomization will be performed by an external data manager who will not be involved in the study intervention or data analysis. Only the team performing the intervention will be informed of the group assignments. The statistician and researchers responsible for data collection will be blinded regarding the group assignments. The inclusion criteria and baseline data of the study will be assessed before the randomization of participants.

### Sample size calculation

The sample size calculation is based on the effect size of the primary outcome, the “mental component” of the HRQoL. A meta-analysis demonstrated an effect size of 2.03 (95% Confidence Interval 1.36 to 2.70) for SWB in support groups but only measured in one single trial [6]. We assume a more conservative effect size of 0.70. This effect size was chosen on the basis of the findings of the study „German Adaptation of REACH II“(GE-REACH) [28], which yielded an effect size between 0.57 (post) and 0.89 (follow-up), thus on average 0.73, for the outcome SWB, measured with mental component summary (MCS) of the General Health Survey Questionnaire Short Form 12 (SF-12). The randomization will be performed on the individual participant level within predefined clusters; a conservative sample size calculation based on a parallel design is computed. A significance level of α of 0.05 (two sided) and a power of 80% for psychological QoL results in a target sample size of 68 participants (34 in each group). An estimated dropout rate of 20% [29] will raise the sample size to 86 study participants. The clusters with eight informal caregivers each lead to a further increase of the sample to 88 informal caregivers (eleven clusters with eight participants each). Therefore, we plan to include 88 informal caregivers in the TALKING TIME study.

### Sample

The sample consists of informal caregivers of people with dementia. They will be recruited for the TALKING TIME study via several public relations strategies (e.g., information folder, articles in journals of health insurance companies, announcements in topic-relevant journals, memory clinics, Alzheimer’s disease associations).

The inclusion criteria for informal caregivers are living or sharing cooking facilities with a person with dementia or providing care for a relative with dementia for at least four hours at least four days a week during the past six months. Moreover, the informal caregivers needed access to a telephone connection to be able to participate in the intervention and the data collection procedure. The care recipient of the informal caregiver must have medical dementia diagnosed based on the criteria of the International Classification of Diseases 10th Revision (ICD-10) [30]: F00.-* = Alzheimer’s disease or related disorders, F01.- = vascular dementia, or F03.- = unspecified dementia.

The exclusion criteria for informal caregivers are a lack of German language skills, an actual psychiatric diagnosis (ICD-10: F10.-*, F20.-*, F00 – F09, F05.-*, F06-*, F08, F09, or F25.-*), and a risk of suicide. A diagnosis in the care recipient of dementia associated with other diseases classified elsewhere (F02.-*), except dementia in primary Parkinson’s disease (F02.3*) and Lewy body disease (F02.8/G31.82), will also be an exclusion criterion. The dementia diagnosis of the care recipient and the presence of a psychiatric disorder or suicidal tendency of the informal caregiver will be explored via telephone and assessed by a psychologist experienced in gerontopsychiatry and psycho-diagnostics. If necessary, an uncertain diagnosis will be clarified with the physician who made the diagnosis by means of a release from confidentiality.

### Intervention

#### Intervention group

The theoretical framework of the telephone-based support groups is based on three theoretical approaches: a) the principles of the theme-centered interaction (TCI) according to Ruth Cohn [31], b) the principles of behavioral therapy [32], and the perspective of systemic therapy [32].

Ad a) The recognition and promotion of the dynamic balance of the ego-we theme-factors (TCI triangle) are the basis of TCI teamwork. Thus, the main task of the support group moderator is to balance these poles by supporting the self-regulation of the group, strengthening the autonomy of group members, and creating an open atmosphere for communication and interaction.

Ad b) These principles are as follows: structured and transparent procedures, clear group structures, emotion, problem- and resource-oriented procedures, and help to self-help.

Ad c) This view may be briefly characterized by the phrase “dementia as a family disease”. This perspective considers role changes, the intra-relationship assumption of new functions and changes in the relationship in general, gradually taking leave from a close relative who is in transition and, simultaneously, increasingly having to turn to this person due to growing dependency.

From the perspective of behavior therapy (b) and systemic therapy (c), the challenging behaviors of people with dementia could be affected by the behavior of the informal caregivers.

The TALKING TIME intervention consists of four fixed components. All components are free of charge and will be implemented as follows:

- ▪ *Component 1*: Telephone-based preliminary talk: To be able to address the concerns of each informal caregiver during the support group sessions (component 3), it is important that the group moderator is aware of the care situation of each individual participating informal caregiver. Therefore, prior to the start of the support groups, the moderator will conduct a preliminary telephone conversation, lasting approximately 30 min, with each informal caregiver. Moreover, the group process, rules of conversation, and a checklist (component 2) are discussed.
- ▪ *Component 2*: Information booklet: To support the thematic introduction of each support group (component 3), each participant will receive an information booklet developed for the TALKING TIME study, summarizing information on the themes (see component 3) of the TALKING TIME sessions and referring to supra-regional contacts and offerings for informal caregivers of people with dementia. The information booklet can be used as a workbook (e.g., for notes) during the support group meeting. The booklet also includes a checklist regarding technical issues (e.g., “what is the battery status of my telephone?”) that must be considered prior to each group session.
- ▪ *Component 3*: Structured telephone-based support groups: Each participant must participate in six telephone-based support group sessions. The support groups are scheduled to occur every two weeks over a three-month period. The groups will consist of a maximum of four informal caregivers and one psychologist who is experienced in working with informal caregivers of people with dementia and who will lead the therapy group as a moderator. The duration of a support group meeting is 60 min. At the beginning of a support group, one of five themes (1. self-care, 2. access to assistance and support offers, 3. communication with healthcare providers (e.g., physicians, qualified nursing staff), 4. communication with family and friends, and 5. improving interaction with the relative with dementia) will be introduced by the moderator. After the thematic introduction, the remaining 45 to 50 min are available for a moderated exchange and discussion among the informal caregivers. At the end of the telephone session, the moderator summarizes the content of the meeting. The content and structure of this telephone-based support group approach are based on the social support component of the caregiver support program Resources for Enhancing Alzheimer’s Caregivers’ Health II (REACH II) [22].
- ▪ *Component 4*: Structured evaluation of each support group session: After each support group session, a structured questionnaire form will be distributed to each support group participant. With this questionnaire, the informal caregivers should individually reflect on each support group session, e.g., “What was good?” “What was my benefit from this session?” “What did I miss, and which questions remain unanswered?” “Which issues do I want to contribute to the next session?”

#### Control group

The informal caregivers of the control group receive none of the components of the TALKING TIME intervention during the intervention phase; during this time, they continue with their usual activities or services, without restriction (usual care). However, to motivate these informal caregivers to participate in the data collection, they will receive all of the components of the TALKING TIME intervention after the T_1_ evaluation has occurred.

### Measurements

To evaluate the intervention effects and to study the process of intervention implementation, quantitative measurements will be applied. The measurements were chosen based on their appropriateness for the target intervention, sample, data collection procedure (telephone interviews), and psychometric properties. Table 1 summarizes all of the measurements. Because not all measurements will be psychometrically tested in the German context, the internal consistency of all applied measurements will be evaluated as part of the descriptive data analysis.

**Table 1:** Measurement instruments

Caregiver				
Variable	Instrument/Source	No. of Items	Measurement	Type of Variable
Self-rated mental health	Mental Component Summary (MCS) of the General Health Survey Questionnaire Short Form 12 (SF-12) [4, 33]	6	T_0_ – T_1_	Primary outcome
Self-rated physical health	Physical Component Summary (PCS) of the General Health Survey Questionnaire Short Form 12 (SF-12) [4, 33]	6	T_0_ – T_1_	Secondary outcome
Social support	Perceived Social Support Caregiving (PSSC) [35]	9	T_0_ – T_1_	Secondary outcome
Social conflict	Perceived Social Conflict Scale (SCS) [35]	3		Control variable
Caregiver reaction	Caregiver Reaction Scale (CRS) [38–40]	24	T_0_ – T_1_	Secondary outcome
Education level	Consortium to Establish a Registry for Alzheimer’s Disease (CERAD) [46]	2	T_0_	Control variable
Demographic variables	Single items	16	T_0_ – T_1_	Control variables
Care Recipient				
Variable	Instrument/Source	No. of Items	Measurement	Type of Variable
Cognition	General Practitioner Assessment of Cognition (GPCOG) [42, 43]	6	T_0_ – T_1_	Control variable
Activities of daily Living	Functional Activities Questionnaire [44, 45]	10	T_0_ – T_1_	Control variable
Challenging behavior	Neuropsychiatric Inventory – Q [41]	12	T_0_ – T_1_	Secondary outcome
Demographic variables	Single items, e.g., age, gender, care dependency (care recipient)	22	T_0_ – T_1_	Control variables
Process Evaluation				
Variable	Instrument (Source)	Measurement		Process Evaluation Domain
Recruitment strategies	Process documents of the TALKING TME study	T_0_		Reach
Numbers of interested, refused, rejected, and included informal caregivers	Process documents of the TALKING TIME study Single items (T0) and process documents of the TALKING TIME study	Ongoing^1^		
Randomization	Process documents of the TALKING TIME study	Ongoing		
Dropouts	Process documents of the TALKING TIME study	Ongoing		
Participant responsiveness	Single items, e.g., quality/satisfaction with the support group content, the group, and its process	T_1_		Fidelity
Quality of delivery	Standardized support group protocols from the support group moderator and single items (T_1_), e.g., quality of the moderator, information booklet, telephone-based preliminary talk	Ongoing		
Adherence	Standardized support group protocols from the support group moderator and single items (T_1_), e.g., deviations from the intervention protocol, usage of the information booklet	Ongoing		Dosage
Dose	Standardized support group protocols from the support group moderator, e.g., participation of the informal caregivers in the support group calls	T_1_		
Socio-demographic characteristics of the participants and care recipients	Single items, e.g., age, gender, care dependency (care recipient)	T_0_ – T_1_		Context
Use of health care services	Use of service checklist [49]	T_0_ – T_1_		

^1^ Ongoing collection after each TALKING TIME support group meeting

### Effect evaluation

The primary outcome is the mental component summary (MCS) of the General Health Survey Questionnaire Short Form 12 (SF-12) [4, 33]. The SF-12 is widely used and has shown its feasibility in telephone interviews [33]. Depending on the items, two, three, five, or six response options are available. The items show different scale levels: two, three, five, or six response categories. The MCS consists of six items that assess mental HRQoL. The total score ranges from 0 to 100.

The secondary outcomes for informal caregivers are self-rated physical HRQoL, social support, social conflict, and caregiver reaction, as well as the proxy-rated challenging behavior of care recipients with dementia. Physical HRQoL will be assessed with the second domain of the SF-12, the physical component summary (PCS). The PCS also consists of six items with scores ranging from 0 to 100. Higher scores of the MCS and the PCS correspond to higher HRQoL values. Both SF-12 component scores have shown adequate reliability and validity [34].

For the assessment of the perceived social support and social conflicts of the informal caregivers, the Perceived Social Support Caregiving instrument (PSSC, 9 items) and Social Conflict Scale (SCS, 3 items) are applied [35]. All items can be answered based on a five-point scale ranging from strongly disagree to strongly agree [36]. Item scores are summed to a total continuous score for social support and social conflict that can range between 9 and 45 and between 3 and 15, respectively. Higher scores indicate a higher level of social support or social conflicts. The original US version has demonstrated sufficient results for reliability and validity [35]. Because the PSSC and SCS were only available in the US English language, we performed a cross-cultural adaptation of both scales, applying an established guideline [37]. During the forward translation, two people independently translated the items and response options into German. Both translators were native German speakers with excellent English language skills. The forward translations were synthesized into one preliminary German version. Two native English speakers, experienced in the translation of instruments and with excellent German language skills, performed the back translation. Both back translators were blinded to the original English version of the scales. Both back-translated versions were compared, and discrepancies were highlighted. All four translators and one additional nurse experienced in the field of dementia care discussed the results and agreed on one final German version for both scales. It was not possible to receive approval from the original author of the scales because contact attempts via e-mail and telephone were unsuccessful. The PSSC results in a total score between 9 and 45, and the SCS yields a total score between 3 and 15. Higher scores indicate more perceived social support and a lower amount of perceived social conflicts, respectively.

Caregiver reaction will be rated with the caregiver reaction scale (CRS) [38–40]. This measurement contains 24 items reflecting caregiver self-esteem (7 items, range: 7 to 35), a lack of family support (5 items, range: 5 to 25), the impact of finances (3 items, range: 3 to 15), the impact of the daily schedule (5 items, range 5 to 25), and the impact on health (4 items, range: 4 to 20). Based on the recommendation of Given et al. [38], we compute the subscale scores, with higher scores indicating a stronger impact. The German version of the CRS has demonstrated sufficient internal consistency and structural validity [39].

The challenging behavior of care recipients will also be assessed as a secondary outcome based on a proxy rating using the Neuropsychiatric Inventory-Q (NPI-Q) [41]. This measurement makes it possible to assess the presence and severity of the following 12 different behaviors and psychological symptoms related to dementia: 1. delusion, 2. hallucination, 3. depression, 4. anxiety, 5. euphoria, 6. aggression, 7. apathy, 8. disinhibition, 9. irritability, 10. aberrant motor behavior, 11. sleep problems, and 12. eating disorders. The severity of the behaviors and psychological symptoms is assessed based on the response options of mild, moderate, and severe. The measurement results in a total score ranging from 0 to 36, with higher scores indicating more challenging behavior. The NPI-Q has shown adequate reliability and validity [41].

Based on a proxy rating given by the informal caregiver, the control variables of cognition and activities of daily living of the care recipient with dementia will be assessed with the General Practitioner Assessment of Cognition (GPCOG) [42, 43] and the Functional Activities Questionnaire (FAQ) [44, 45]. The six items of the proxy version of the GPCOG can be completed based on the response options of yes, no, and do not know. The total score ranges between 0 and 6, with higher scores indicating better cognition [42]. The four response options of the 10 FAQ items are normal, has difficulty but does by self, requires assistance, and dependent. The total score ranges from 0 to 30, with higher scores indicating a higher impaired function. The FAQ has demonstrated sufficient reliability and validity [45]. Socio-demographic data, e.g., age, gender of the informal caregiver and the care recipient with dementia, and care dependency level as defined by German long-term care insurance of the person with dementia, will be rated with single items. The educational level of the informal caregiver will be assessed according to a procedure used in the Consortium to Establish a Registry for Alzheimer’s Disease (CERAD) [46].

To ensure standardization, the data collection will be performed in a telephone interview. Each participating informal caregiver will receive a printed TALKING TIME questionnaire with all measures and items prior to the telephone interview. The actual interviews will be initiated by members of the research team, who are registered nurses and academically qualified nursing researchers experienced in data collection procedures in dementia research. A comprehensive instruction manual regarding data collecting and data handling will be provided to support each interviewer.

### Process evaluation

Data for the process evaluation will be collected continuously throughout the study and as part of the telephone interviews at both measurement points (T_0_, T_1_) to investigate the process evaluation domains of *reach, fidelity, dosage,* and *context* [47, 48]. Different data sources will be used to evaluate the recruitment and intervention process (see Table 1).

Within the *reach* domain, variables reflecting the recruitment process, like the recruitment strategy, the numbers of interested, refused, rejected, and included individuals, the randomization, and the number and reasons for dropouts, will be collected. Intervention *fidelity* will be assessed with single items during the telephone interviews at the T_1_ measurement point. The single items reflect, e.g., the quality of the moderator, the information booklet, the preparatory calls, the group process, schedule difficulties, and technical problems during the telephone-based support groups. Moreover, the structured evaluation of each support group session (questionnaire) by each study participant is one further potential data source for the investigation of intervention fidelity.

The intervention *dosage* will be investigated based on data on the adherence to the protocol and actual attendance of the informal caregivers. These data will be collected with standardized protocols for each support group session completed by the moderator and single items rated during the telephone interviews.

The *context* of the intervention implementation will be evaluated using the data on the characteristics of the study participants and their care recipients. Moreover, the use and non-use of health care services and the associated reasons for their use or non-use will be assessed with the standardized use of service checklist [49]. The checklist contains 24 items and was developed as part of the European Actifcare study. Each of the items can be answered based on 12 different response options reflecting the use and non-use and associated reasons [50].

## Data analysis

### Effect analysis

The primary and secondary outcomes from the intervention and control groups will be compared using descriptive and advanced statistical methods at T_0_ and T_1_. A mixed effect model will be computed to evaluate the differences between the intervention and control groups regarding the development of primary and all secondary outcomes during the study period (T_1_). The mixed effect model will account for the repeated measurements of the participants. Additionally, a model for evaluating the impact of the between cluster differences will be tested by including the clusters as random effects. A cluster is defined as a group of a maximum of four informal caregivers who represent the members of one support group. To evaluate the effect of the assignment, the intention to treat principle (ITT) will be applied. The results of the ITT will be compared to a per protocol analysis to assess the effect of intervention adherence. In all analytical steps, the effect estimates will be adjusted for baseline differences and differences in the control variables at all measurement points. The mixed effect model for the primary outcome will also consider the secondary outcomes as control variables. A statistician who is blinded to group allocation will perform the statistical analyses. The research team members responsible for the effect evaluation will be blinded to the results of the process evaluation until the final mixed effect model for the primary outcome is computed.

### Process analysis

The process evaluation data will be analyzed using descriptive statistical methods. Following an analysis of the data set of each intervention cluster, the results for each cluster will be synthesized in a side by side comparison. The members of the research team responsible for the process evaluation will be blinded to the effectiveness results until the completion of the process evaluation for the first three clusters.

### Study progress

The study design and protocol were approved by the Ethics Committee of the University of Leipzig in February 2016 (Register number: 052/16-ek). The recruitment began in March 2016 and collection of informed consent began in June 2016. The assessment of baseline data for the first support group cluster began in July 2016. The last T_1_ measurement, effect, and process evaluations are scheduled for March 2017. Analysis of the data and the dissemination of the results are planned for summer 2017.

## Discussion

This randomized controlled trial will provide important information on the effectiveness of the TALKING TIME intervention as a structured telephone-based support group for informal caregivers of people with dementia and knowledge on the effective and ineffective intervention components and trial procedures (e.g., sample recruitment).

Previous studies investigating supportive interventions for informal caregivers have demonstrated no risks for participants (Dam et al. [9]). However, based on the measurement of the primary and secondary outcomes for informal caregivers and people with dementia, as well as the process evaluation, this trial allows a comprehensive evaluation of the types of intervention harm. In summary, the study findings will be an important basis for the design of a subsequent randomized controlled trial in the third phase of the MRC framework [27].

## Abbreviations

HRQoL: Health-related quality of life
QoL: Quality of life
SWB: Subjective Wellbeing
